# Acne Scars: Pathogenesis, Classification and Treatment

**DOI:** 10.1155/2010/893080

**Published:** 2010-10-14

**Authors:** Gabriella Fabbrocini, M. C. Annunziata, V. D'Arco, V. De Vita, G. Lodi, M. C. Mauriello, F. Pastore, G. Monfrecola

**Affiliations:** Division of Clinical Dermatology, Department of Systematic Pathology, University of Naples Federico II, Via Sergio Pansini 5, 80133 Napoli, Italy

## Abstract

Acne has a prevalence of over 90% among adolescents and persists into adulthood in approximately 12%–14% of cases with psychological and social implications. Possible outcomes of the inflammatory acne lesions are acne scars which, although they can be treated in a number of ways, may have a negative psychological impact on social life and relationships. The main types of acne scars are atrophic and hypertrophic scars. The pathogenesis of acne scarring is still not fully understood, but several hypotheses have been proposed. There are numerous treatments: chemical peels, dermabrasion/microdermabrasion, laser treatment, punch techniques, dermal grafting, needling and combined therapies for atrophic scars: silicone gels, intralesional steroid therapy, cryotherapy, and surgery for hypertrophic and keloidal lesions. This paper summarizes acne scar pathogenesis, classification and treatment options.

## 1. Introduction

Acne has a prevalence of over 90% among adolescents [1] and persists into adulthood in approximately 12%–14% of cases with psychological and social implications of high gravity [2, 3]. 

All body areas with high concentrations of pilosebaceous glands are involved, but in particular the face, back and chest. Inflammatory acne lesions can result in permanent scars, the severity of which may depend on delays in treating acne patients. The prevalence and severity of acne scarring in the population has not been well studied, although the available literature is usually correlated to the severity of acne [4]. 2133 volunteers aged 18 to 70 from the general population showed that nearly 1% of people had acne scars, although only 1 in 7 of these were considered to have “disfiguring scars” [5]. Severe scarring caused by acne is associated with substantial physical and psychological distress, particularly in adolescents.

## 2. Pathogenesis

The pathogenesis of acne is currently attributed to multiple factors, such as increased sebum production, alteration of the quality of sebum lipids, androgen activity, proliferation of Propionibacterium acnes (P. acnes) within the follicle and follicular hyperkeratinization [6]. Increased sebum excretion contributes to the development of acne. Neutral and polar lipids produced by sebaceous glands serve a variety of roles in signal transduction and are involved in biological pathways [7]. Additionally, fatty acids act as ligands of nuclear receptors such as PPARs. Sebaceous gland lipids exhibit direct pro- and anti-inflammatory properties, whereas the induction of 5-lipoxygenase and cyclooxygenase-2 pathways in sebocytes leads to the production of proinflammatory lipids [8]. Furthermore, hormones like androgens control sebaceous gland size and sebum secretion. In cell culture, androgens only promote sebocyte proliferation, whereas PPAR ligands are required for the induction of differentiation and lipogenic activity [9]. On the other hand, keratinocytes and sebocytes may be activated by P. acnes via TLR, CD14, and CD1 molecules [10]. Pilosebaceous follicles in acne lesions are surrounded by macrophages expressing TLR2 on their surface. TLR2 activation leads to a triggering of the transcription nuclear factor and thus the production of cytokines/chemokines, phenomena observed in acne lesions. Furthermore, P. acnes induces IL-8 and IL-12 release from TLR2 positive monocytes [11]. 

All these events stimulate the infrainfundibular inflammatory process, follicular rupture, and perifollicular abscess formation, which stimulate the wound healing process. Injury to the skin initiates a cascade of wound healing events. Wound healing is one of the most complex biological process and involves soluble chemical mediators, extracellular matrix components, parenchymal resident cells as keratinocytes, fibroblasts, endothelial cells, nerve cells, and infiltrating blood cells like lymphocytes, monocytes, and neutrophils, collectively known as immunoinflammatory cells. Scars originate in the site of tissue injury and may be atrophic or hypertrophic. The wound healing process progresses through 3 stages: (1) inflammation, (2) granulation tissue formation, and (3) matrix remodeling [12, 13].

1. *Inflammation*. Blanching occurs secondary to vasoconstriction for hemostasis. After the blood flow has been stopped, vasodilatation and resultant erythema replace vasoconstriction. Melanogenesis may also be stimulated. This step plays an important role in the development of postacne erythema and hyperpigmentation. A variety of blood cells, including granulocytes, macrophages, neutrophils lymphocytes, fibroblasts, and platelets, are activated and release inflammatory mediators, which ready the site for granulation tissue formation [14]. By examining biopsy specimens of acne lesions from the back of patients with severe scars and without scars, Holland et al. found that the inflammatory reaction at the pilosebaceous gland was stronger and had a longer duration in patients with scars versus those without; in addition, the inflammatory reaction was slower in those with scars versus patients who did not develop scars. They showed a strong relationship between severity and duration of inflammation and the development of scarring, suggesting that treating early inflammation in acne lesions may be the best approach to prevent acne scarring [15].
2. *Granulation Tissue Formation*. Damaged tissues are repaired and new capillaries are formed. Neutrophils are replaced by monocytes that change into macrophages and release several growth factors including platelet-derived growth factor, fibroblast growth factor, and transforming growth factors *α* and *β*, which stimulate the migration and proliferation of fibroblasts [16]. New production of collagen by fibroblasts begins approximately 3 to 5 days after the wound is created. Early on, the new skin composition is dominated by type III collagen, with a small percentage (20%) of type I collagen. However, the balance of collagen types shifts in mature scars to be similar to that of unwounded skin, with approximately 80% of type I collagen [17].
3. *Matrix Remodelling*. Fibroblasts and keratinocytes produce enzymes including those that determine the architecture of the extracellular matrix metalloproteinases (MMPs) and tissue inhibitors of MMPs. MMPs are extracellular matrix (ECM) degrading enzymes that interact and form a lytic cascade for ECM remodeling [18]. As a consequence, an imbalance in the ratio of MMPs to tissue inhibitors of MMPs results in the development of atrophic or hypertrophic scars. Inadequate response results in diminished deposition of collagen factors and formation of an atrophic scar while, if the healing response is too exuberant, a raised nodule of fibrotic tissue forms hypertrophic scars [19].

## 3. Morphology, Histology, and Classification

Scarring can occur as a result of damage to the skin during the healing of active acne. There are two basic types of scar depending on whether there is a net loss or gain of collagen (atrophic and hypertrophic scars). Eighty to ninety percent of people with acne scars have scars associated with a loss of collagen (atrophic scars) compared to a minority who show hypertrophic scars and keloids. 

### 3.1. Atrophic Scars

**Figure 1:**
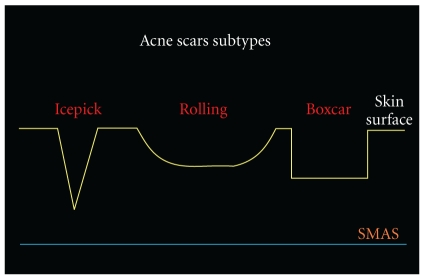
Acne scars subtypes.

Atrophic acne scars are more common than keloids and hypertrophic scars with a ratio 3 : 1. They have been subclassified into ice pick, boxcar, and rolling scars (Figure 1 and Table 1). With atrophic scars, the ice pick type represents 60%–70% of total scars, the boxcar 20%–30%, and rolling scars 15%–25% [20].

**Figure 2:**
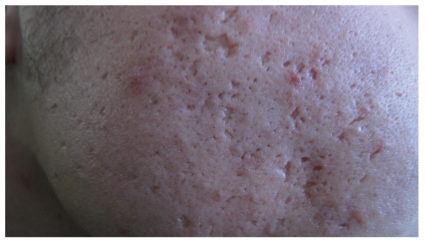
Icepick scars.

Icepick: narrow (2 mm), punctiform, and deep scars are known as icepick scars. With this type of scar, the opening is typically wider than the deeper infundibulum (forming a ‘‘V” shape) (Figure 2). 

**Figure 3:**
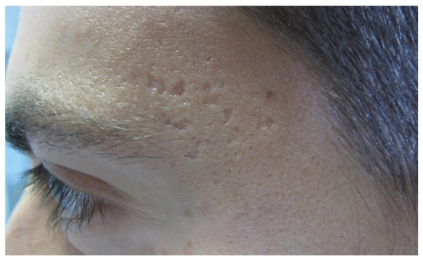
Boxcar scars.

Rolling: dermal tethering of the dermis to the subcutis characterizes rolling scars, which are usually wider than 4 to 5 mm. These scars give a rolling or undulating appearance to the skin (‘‘M” shape). Boxcar: round or oval scars with well-established vertical edges are known as boxcar scars. These scars tend to be wider at the surface than an icepick scar and do not have the tapering V shape. Instead, they can be visualized as a ‘‘U” shape with a wide base. Boxcar scars can be shallow or deep (Figure 3). 

Sometimes the 3 different types of atrophic scars can be observed in the same patients and it can be very difficult to differentiate between them. For this reason several classifications and scales have been proposed by other authors. Goodman and Baron proposed a qualitative scale and then presented a quantitative scale [21, 22]. Dreno et al. introduced the ECCA scale (Echelle d'Evaluation Clinique des Cicatrices d'Acné) [23]. 

The qualitative scarring grading system proposed by Goodman and Baron [9] is simple and universally applicable. According to this classification, four different grades can be used to identify an acne scar, as shown in Table 2. Often (especially in those affected with mild acne) the pattern and grading is easy to achieve but, in the observation of severe cases, different patterns are simultaneously present and may be difficult to differentiate. The standard approach adopted by Goodman and Baron describes a grading pattern and they developed a quantitative global acne scarring assessment tool [22] based on the type of scar and the number of scars. This system assigns fewer points to macular and mild atrophic scores than to moderate and severe atrophic scores (macular or mildly atrophic: 1 point; moderately atrophic: 2 points; punched out or linear-troughed severe scars: 3 points; hyperplastic papular scars: 4 points). The multiplication factor for these lesion types is based on the numerical range whereby, for one to ten scars, the multiplier is 1; for 11–20 it is 2; for more than 20 it is 3. 

The ECCA (Echelle d'Evaluation clinique des Cicatrices d'acné) for facial acne scarring is also a quantitative scale, designed for use in clinical practice with the aim of standardizing discussion on scar treatment and it is based on the sum of individual types of scars and their numerical extent. Scar types considered to be more visibly disfiguring were weighted more heavily. Specific scar types and their associated weighting factors were the following: atrophic scars with diameter less than 2 mm: 15; U-shaped atrophic scars with a diameter of 2–4 mm: 20; M-shaped atrophic scars with diameter greater than 4 mm: 25; superficial elastolysis: 30; hypertrophic scars with a less than 2-year duration: 40; hypertrophic scars of greater than 2-year duration: 50. A semiquantitative assessment of the number of each of these scar types was then determined with a four-point scale, in which 0 indicates no scars, 1 indicates less than five scars, 2 indicates between five and 20 scars, and 3 indicates more than 20 scars. With this method, the relative extent of scarring for each scar type was calculated. The total score can vary from 0 to 540. In recent studies on the reliability of this scale, seven dermatologists underwent a 30-min training session prior to the evaluation of ten acne patients. There was no statistical difference in score grading between participating dermatologists. The global scores, however, varied from a minimum of 15 to a maximum of 145. Unfortunately, a statistical estimate of reliability within and between raters was not provided. The potential advantages of this system include independent accounting of specific scar types, thereby providing for separate atrophic and hypertrophic subscores in addition to total scores. Potential shortcomings include restriction to facial involvement, time intensity, and undetermined clinical relevance of score ranges [21].

### 3.2. Hypertrophic and Keloidal Scars

Hypertrophic and keloidal scars are associated with excess collagen deposition and decreased collagenase activity. Hypertrophic scars are typically pink, raised, and firm, with thick hyalinized collagen bundles that remain within the borders of the original site of injury. The histology of hypertrophic scars is similar to that of other dermal scars. In contrast, keloids form as reddish-purple papules and nodules that proliferate beyond the borders of the original wound; histologically, they are characterized by thick bundles of hyalinized acellular collagen arranged in whorls. Hypertrophic and keloidal scars are more common in darker-skinned individuals and occur predominantly on the trunk. 

## 4. Treatment

New acquisitions by the literature have showed that prevention is the main step in avoiding the appearance of post-acne scars. Genetic factors and the capacity to respond to trauma are the main factors influencing scar formation [24]. A number of treatments are available to reduce the appearance of scars. First, it is important to reduce as far as possible the duration and intensity of the inflammation, thus stressing the importance of the acne treatment. The use of topical retinoids is useful in the prevention of acne scars but more than any other measure, the use of silicone gel has a proven efficacy in the prevention of scars, especially for hypertrophic scars and keloids.

### 4.1. Atrophic Scars

#### 4.1.1. Chemical Peels

By chemical peeling we mean the process of applying chemicals to the skin to destroy the outer damaged layers and accelerate the repair process [25].

Chemical peeling is used for the reversal of signs of skin aging and for the treatment of skin lesions as well as scars, particularly acne scars. Dyschromias, wrinkles, and acne scars are the major clinical indications for facial chemical peeling [26, 27]. 

As regards acne scars, the best results are achieved in macular scars. Icepick and rolling scars cannot disappear completely and need sequential peelings together with homecare treatment with topical retinoids and alpha hydroxy acids [28, 29]. The level of improvement expected is extremely variable in different diseases and patients. For example, ice pick acne scars in a patient with hyperkeratotic skin are only mildly improved even if skin texture is remodeled. On the other hand, a patient with isolated box scars can obtain a significant improvement by application of TCA at 50%–90% on the single scars. 

Several hydroxy acids can be used.

**(A) Glycolic Acid**

Glycolic acid is an alpha-hydroxy acid, soluble in alcohol, derived from fruit and milk sugars. Glycolic acid acts by thinning the stratum corneum, promoting epidermolysis and dispersing basal layer melanin. It increases dermal hyaluronic acid and collagen gene expression by increasing secretion of IL-6 [30]. The procedure is well tolerated and patient compliance is excellent, but glycolic acid peels are contraindicated in contact dermatitis, pregnancy, and in patients with glycolate hypersensitivity. Side effects, such as temporary hyperpigmentation or irritation, are not very significant [31]. Some studies showed that the level of skin damage with glycolic acid peel increases in a dose- and time-dependent manner. The acid at the higher concentration (70%) created more tissue damage than the acid at the lower concentration (50%) compared to solutions with free acid. An increase in the transmembrane permeability coefficient is observed with a decrease in pH, providing a possible explanation for the effectiveness of glycolic acid in skin treatment [32]. The best results achieved for acne scars regard five sequential sessions of 70% glycolic acid every 2 weeks.

**(B) Jessner's Solution**

Formulated by Dr. Max Jessner, this combination of salicylic acid, resorcinol, and lactic acid in 95% ethanol is an excellent superficial peeling agent. Resorcinol is structurally and chemically similar to phenol. It disrupts the weak hydrogen bonds of keratin and enhances-penetration of other agents [33]. Lactic acid is an alpha hydroxy acid which causes corneocyte detachment and subsequent desquamation of the stratum corneum [34]. As with other superficial peeling agents, Jessner's peels are well tolerated. General contraindications include active inflammation, dermatitis or infection of the area to be treated, isotretinoin therapy within 6 months of peeling and delayed or abnormal wound healing. Allergic contact dermatitis and systemic allergic reactions to resorcinol are rare and need to be considered as absolute contraindications [35, 36].

**(C) Pyruvic Acid**

Pyruvic acid is an alpha-ketoacid and an effective peeling agent [37]. It presents keratolytic, antimicrobial and sebostatic properties as well as the ability to stimulate new collagen production and the formation of elastic fibers [38]. The use of 40%–70% pyruvic acid has been proposed for the treatment of moderate acne scars [39, 40]. Side effects include desquamation, crusting in areas of thinner skin, intense stinging, and a burning sensation during treatment. Pyruvic acid has stinging and irritating vapors for the upper respiratory mucosa, and it is advisable to ensure adequate ventilation during application.

**(D) Salicylic Acid**

Salicylic acid is one of the best peeling agents for the treatment of acne scars [41]. It is a beta hydroxy acid agent which removes intercellular lipids that are covalently linked to the cornified envelope surrounding cornified epithelioid cells. The most efficacious concentration for acne scars is 30% in multiple sessions, 3–5 times, every 3-4 weeks [42–44]. The side effects of salicylic acid peeling are mild and transient. These include erythema and dryness. Persistent postinflammatory hyperpigmentation or scarring are very rare and for this reason it is used to treat dark skin [45]. Rapid breathing, tinnitus, hearing loss, dizziness, abdominal cramps, and central nervous system symptoms characterize salicylism or salicylic acid toxicity. This has been observed with 20% salicylic acid applied to 50% of the body surface [46]. Grimes has peeled more than 1,000 patients with the current 20 and 30% marketed ethanol formulations and has observed no cases of salicylism [47].

**(E) Trichloroacetic Acid**

The use of trichloroacetic acid (TCA) as a peeling agent was first described by P.G. Unna, a German dermatologist, in 1882. TCA application to the skin causes protein denaturation, the so-called keratocoagulation, resulting in a readily observed white frost [48]. For the purposes of chemical peeling, it is mixed with 100 mL of distilled water to create the desired concentration. The degree of tissue penetration and injury by a TCA solution is dependent on several factors, including percentage of TCA used, anatomic site, and skin preparation. Selection of appropriate TCA-concentrated solutions is critical when performing a peel. TCA in a percentage of 10%–20% results in a very light superficial peel with no penetration below the stratum granulosum; a concentration of 25%–35% produces a light superficial peel with diffusion encompassing the full thickness of the epidermis; 40%–50% can produce injury to the papillary dermis; and finally, greater than 50% results in injury extending to the reticular dermis. Unfortunately the use of TCA concentrations above 35% TCA can produce unpredictable results such as scarring. Consequently, the medium depth chemical peel should only be obtained with the combination of 35% TCA. The use of TCA in concentrations greater than 35%, should be avoided. It can be preferred in some cases of isolated lesions or for treatment of isolated icepick scars (TCA CROSS) [49]. When performed properly, peeling with TCA can be one of the most satisfying procedures in acne scar treatment but it is not indicated for dark skin because of the high risk of hyperpigmentation [50].

**(F) TCA Cross**

**Figure 4:**
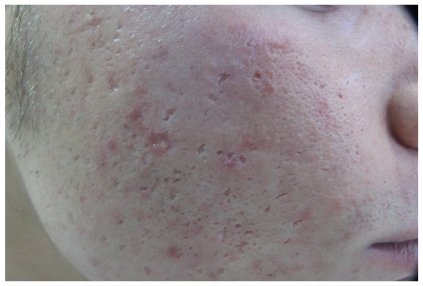
TCA Cross: patient before the treatment.

**Figure 5:**
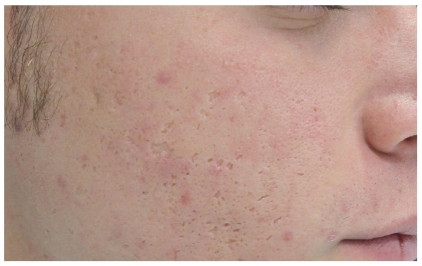
TCA Cross: patient after the treatment.

In our experience the TCA CROSS technique has shown high efficacy in the case of few isolated scars on healthy skin. CROSS stands for chemical reconstruction of skin scars method and involves local serial application of high concentration TCA to skin scars with wooden applicators sized via a number 10 blade to a dull point to approximate the shape of the scar. No local anesthesia or sedation is needed to perform this technique [51]. Unlike the reports found in the literature, in which 90% TCA is suggested, our experiences have shown that a lower TCA concentration (50%) has similar results and much less adverse reactions [52]. TCA is applied for a few seconds until the scar displays a white frosting. Emollients then needs to be prescribed for the following 7 days and high photoprotection is required. The procedure should be repeated at 4-week intervals, and each patient receives a total of three treatments. Our experiences have shown that, compared with other procedures, this technique can avoid scarring and reduce the risk of hypopigmentation by sparing the adjacent normal skin and adnexal structures [53] (Figures 4 and 5).

#### 4.1.2. Dermabrasion/Microdermabrasion

Dermabrasion and microdermabrasion are facial resurfacing techniques that mechanically ablate damaged skin in order to promote reepithelialisation. Although the act of physical abrasion of the skin is common to both procedures, dermabrasion, and microdermabrasion employ different instruments with a different technical execution [54]. Dermabrasion completely removes the epidermis and penetrates to the level of the papillary or reticular dermis, inducing remodeling of the skin's structural proteins. Microdermabrasion, a more superficial variation of dermabrasion, only removes the outer layer of the epidermis, accelerating the natural process of exfoliation [55, 56]. Both techniques are particularly effective in the treatment of scars and produce clinically significant improvements in skin appearance. Dermabrasion is performed under local or general anaesthesia. A motorized hand piece rotates a wire brush or a diamond fraise. Several decades ago, the hand piece was made of aluminum oxide or sodium bicarbonate crystals, whereas now diamond tips have replaced these hand pieces to increase accuracy and decrease irritation. There is often a small pinpoint bleeding of the raw wound that subsides with appropriate wound care. Patients with darker skin may experience permanent skin discoloration or blotchiness. As regards the technique of microdermabrasion, a variety of microdermabraders are available. All microdermabraders include a pump that generates a stream of aluminum oxide or salt crystals with a hand piece and vacuum to remove the crystals and exfoliate the skin [57]. Unlike dermabrasion, microdermabrasion can be repeated at short intervals, is painless, does not require anesthesia and is associated with less severe and rare complications, but it also has a lesser effect and does not treat deep scars [58, 59].

It is essential to conduct a thorough investigation of the patient's pharmacological history to ensure that the patient has not taken isotretinoin in the previous 6–12 months. As noted by some studies [60], the use of tretinoin causes delayed reepithelialization and development of hypertrophic scars.

#### 4.1.3. Laser Treatment

All patients with box-car scars (superficial or deep) or rolling scars are candidates for laser treatment. Different types of laser, including the nonablative and ablative lasers are very useful in treating acne scars. Ablative lasers achieve removal of the damaged scar tissue through melting, evaporation, or vaporization. Carbon dioxide laser and Erbium YAG laser are the most commonly used ablative lasers for the treatment of acne scars. These abrade the surface and also help tighten the collagen fibers beneath. Nonablative lasers do not remove the tissue, but stimulate new collagen formation and cause tightening of the skin resulting in the scar being raised to the surface. Among the nonablative lasers the most commonly used are the NdYAG and Diode lasers [61].

The ablative lasers are technologies with a high selectivity for water. Therefore, their action takes place mainly on the surface but the depth of action is certainly to be correlated to the intensity of the emitted energy and the diameter of the spot used. Among the ablative lasers, Erbium technologies are so selective for water that their action is almost exclusively ablative. CO_2_ lasers, which present lower selectivity for water, besides causing ablation are also capable of determining a denaturation in the tissues surrounding the ablation and a thermal stimulus not coagulated for dermal protein. CO_2_ lasers have a double effect: they promote the wound healing process and arouse an amplified production of myofibroblasts and matrix proteins such as hyaluronic acid [62].

Clinical and histopathologic studies have previously demonstrated the efficacy of CO_2_ laser resurfacing in the improvement of facial atrophic acne scars, with a 50%–80% improvement typically seen. The differences in results reported with apparently similar laser techniques may be due to variations in the types of scar treated. Candidates must present a skin disease with acne off for at least 1 year; they should have stopped taking oral isotretinoin for at least 1 year; they should not have presented skin infections by herpes virus during the six months prior to treatment; they must not have a history of keloids or hypertrophic scarring. Patients with a high skin type phototype are exposed to a higher risk of hyperpigmentation after treatment than patients with low phototype.

All ablative lasers showed high risk of complications and side effects. Adverse reactions to the first generation of ablative lasers can be classified into short-term (bacterial, herpetic or fungal infections) and long-term (persistent erythema, hyperpigmentation, scarring) [63, 64]. In particular, scarring after CO_2_ laser therapy may be due to the over treatment of the areas (including excessive energy, density, or both), lack of technical aspects, infection, or idiopathic. It is necessary to take into account these aspects when sensitive areas such as the eyelids, upper neck, and especially the lower neck and chest are treated [65, 66]. 

Nonablative skin remodeling systems have become increasingly popular for the treatment of facial rhytides and acne scars because they decrease the risk of side effects and the need for postoperative care. Nonablative technology using long-pulse infrared (1.450 nm diode, 1320 and 1064 nm neodymium-doped yttrium aluminum garnet (Nd:YAG), and 1540 nm erbium glass) was developed as a safe alternative to ablative technology for inducing a controlled thermal injury to the dermis, with subsequent neocollagenesis and remodeling of scarred skin [67–72]. 

Although improvement was noted with these nonablative lasers, the results obtained were not as impressive as the results from those using laser resurfacing [71]. 

For this reason, a new concept in skin laser therapy, called fractional photothermolysis, has been designed to create microscopic thermal wounds to achieve homogeneous thermal damage at a particular depth within the skin, a method that differs from chemical peeling and laser resurfacing. Prior studies using fractional photothermolysis have demonstrated its effectiveness in the treatment of acne scars [73] with particular attention for dark skin to avoid postinflammatory hyperpigmentation [74]. 

Newer modalities using the principles of fractional photothermolysis devices (FP) to create patterns of tiny microscopic wounds surrounded by undamaged tissues are new devices that are preferred for these treatments. These devices produce more modest results in many cases than traditional carbon dioxide lasers but have few side effects and short recovery periods [75]. Many fractional lasers are available with different types of source. A great deal of experience with nonablative 1550 nm erbium doped fractional photothermolysis has shown that the system can be widely used for clinical purposes. 

An ablative 30 W CO_2_ laser device uses ablative fractional resurfacing (AFR) and combines CO_2_ ablation with an FP system. By depositing a pixilated pattern of microscopic ablative wounds surrounded by healthy tissue in a manner similar to that of FP [76], AFR combines the increased efficacy of ablative techniques with the safety and reduced downtime associated with FP.

Topographic analysis performed by some authors has shown that the depth of acneiform scars has quantifiable objective improvement ranging from 43% to 80% with a mean level of 66.8% [77]. The different experiences of numerous authors in this field have shown that, by combining ablative technology with FP, AFR treatments constitute a safe and effective treatment modality for acneiform scarring. Compared to conventional ablative CO_2_ devices the side effects profile is greatly improved and, as with FP, rapid reepithelization from surrounding undamaged tissue is believed to be responsible for the comparatively rapid recovery and reduced downtime noted with AFR [78–80]. 

Pigmentation abnormalities following laser treatment is always a concern. Alster and West reported 36% incidence of hyperpigmentation when using conventional CO_2_ resurfacing compared to a minority of patients treated with AFR treatments, probably linked to shortened period of recovery and posttreatment erythema [81]. The treatment strategy is linked to establishing the optimal energy, the interval between sessions, and a longer follow-up period to optimize treatment parameters.

#### 4.1.4. Punch Techniques

Atrophic scarring is the more common type of scarring encountered after acne. Autologous and nonautologous tissue augmentation, and the use of punch replacement techniques has added more precision and efficacy to the treatment of these scars [82].

The laser punch-out method is better than even depth resurfacing for improving deep acne scars and can be combined with the shoulder technique or even depth resurfacing according to the type of acne scar [83]. 

Laser skin resurfacing with the concurrent use of punch excision improves facial acne scarring [84].

#### 4.1.5. Dermal Grafting

Acne scars may be treated surgically using procedures such as dermabrasion and/or simple scar excision, scar punch elevation, or punch grafting [85].

The useful modalities available are dermal punch grafting, excision, and facelifting. The selection of these techniques is dependent on the above classification and the patient's desire for improvement [86].

Split-thickness or full-thickness grafts “take" on a bed of scar tissue or dermis following the removal of the epidermis. The technique is useful in repairing unstable scars from chronic leg ulcers or X-ray scars. It can also camouflage acne scars, extensive nevi pigmentosus, and tattoos [87, 88]. It is prepackaged dermal graft material that is easy to use, safe, and effective [89].

#### 4.1.6. Tissue Augmenting Agents

*Fat transplantation. *Fat is easily available and it has low incidence of side effects [90]. The technique consists of two phases: procurement of the graft and placement of the graft. The injection phase with small parcels of fat implanted in multiple tunnels allows the fat graft maximal access to its available bloody supply. The fat injected will normalize the contour excepted where residual scar attachments impede this.

#### 4.1.7. Other Tissue Augmenting Agents

There are many new and older autologous, nonautologous biologic, and nonbiologic tissue augmentation agents that have been used in the past for atrophic scars, such as autologous collagen, bovine collagen, isolagen, alloderm, hyaluronic acid, fibrel, artecoll, and silicon, but nowadays, because of the high incidence of side effects, the recommended material to use is hyaluronic acid [91].

#### 4.1.8. Needling

**Figure 6:**
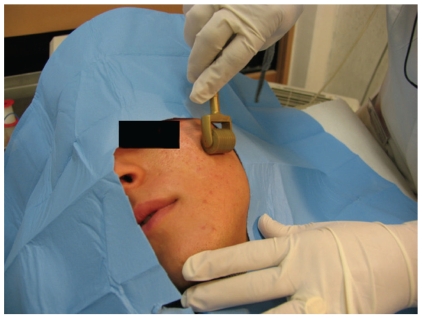
Needling: the procedure.

**Figure 7:**
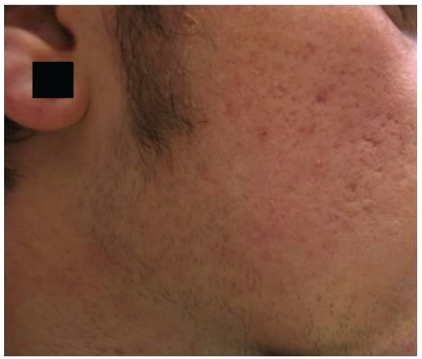
Needling: patient before the treatment.

**Figure 8:**
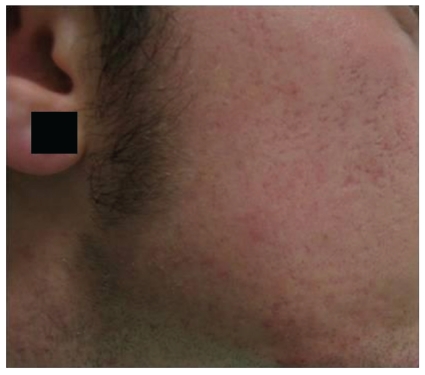
Needling: patient after the treatment.

Skin needling is a recently proposed technique that involves using a sterile roller comprised of a series of fine, sharp needles to puncture the skin. At first, facial skin must be disinfected, then a topical anesthetic is applied, left for 60 minutes. The skin needling procedure is achieved by rolling a performed tool on the cutaneous areas affected by acne scars (Figure 6), backward and forward with some pressure in various directions. The needles penetrate about 1.5 to 2 mm into the dermis. As expected, the skin bleeds for a short time, but that soon stops. The skin develops multiple microbruises in the dermis that initiate the complex cascade of growth factors that finally results in collagen production. Histology shows thickening of skin and a dramatic increase in new collagen and elastin fibers. Results generally start to be seen after about 6 weeks but the full effects can take at least three months to occur and, as the deposition of new collagen takes place slowly, the skin texture will continue to improve over a 12 month period. Clinical results vary between patients, but all patients achieve some improvements (Figures 7 and 8). The number of treatments required varies depending on the individual collagen response, on the condition of the tissue and on the desired results. Most patients require around 3 treatments approximately 4 weeks apart. Skin needling can be safely performed on all skin colours and types: there is a lower risk of postinflammatory hyperpigmentation than other procedures, such as dermabrasion, chemical peelings, and laser resurfacing. Skin needling is contraindicated in the presence of anticoagulant therapies, active skin infections, collagen injections, and other injectable fillers in the previous six months, personal or familiar history of hypertrophic and keloidal scars [92, 93].

#### 4.1.9. Combined Therapy

There is a new combination therapy for the treatment of acne scars. The first therapy consists of peeling with trichloroacetic acid, then followed by subcision, the process by which there is separation of the acne scar from the underlying skin and in the end fractional laser irradiation. The efficacy and safety of this method was investigated for the treatment of acne scars. The duration of this therapy is 12 months. Dot peeling and subcision were performed twice 2-3 months apart and fractional laser irradiation was performed every 3-4 weeks. There were no significant complications at the treatment sites. It would appear that triple combination therapy is a safe and very effective combination treatment modality for a variety of atrophic acne scars [94].

### 4.2. Hypertrophic Scars

#### 4.2.1. Silicone Gel

Silicone-based products represent one of the most common and effective solutions in preventing and also in the treatment of hypertrophic acne scars. The silicone gel was introduced in the treatment of hypertrophic acne scars to overcome the difficulties in the management of silicone sheets. Indeed, the silicone gel has several advantages: it is transparent, quick drying, nonirritating and does not induce skin maceration, it can be used to treat extensive scars and uneven areas of skin. The mechanism of action is not fully understood but several hypotheses [95] have been advanced: (1) the increase in hydration; (2) the increase in temperature; (3) protection of the scar; (4) increased tension of O2; (5) action on the immune system. There is, currently, only one observational open label study, conducted on 57 patients. In this study, the gel was applied on the scars 2 times daily for 8 weeks with an average improvement in the thickness estimated between 40% and 50% compared to baseline. 

As regards the treatment of already formed hypertrophic scars, the gel should be applied in small amounts, twice daily for at least 8 weeks to achieve a satisfactory aesthetic result. Whereas for the purposes of prevention, the same dosage is recommended for at least 12–16 weeks; the treatment should be started as soon as possible after the risk of a patient developing hypertrophic acne scars has been identified.

Treatment with silicone gel can be used in patients of any age and women of childbearing age. Moreover, the silicone gel can be used throughout the year, including summer.

#### 4.2.2. Intralesional Steroid Therapy

Intralesional injection of steroids is one of the most common treatments for keloids and hypertrophic scars. It can be used alone or as part of multiple therapeutic approaches. Corticosteroids may reduce the volume, thickness, and texture of scars, and they can relieve symptoms such as itching and discomfort [96]. The mechanisms of action have not been completely clarified: in addition to their anti-inflammatory properties, it has been suggested that steroids exert a vasoconstrictor and an antimitotic activity. It is believed that steroids arrest pathological collagen production through two distinct mechanisms: the reduction of oxygen and nutrients to the scar with inhibition of the proliferation of keratinocytes and fibroblasts [96]; the stimulation of digestion of collagen deposition through block of a collagenase-inhibitor, the alpha-2-microglobulin [97]. During the injection the syringe needle should be kept upright [24]. It is always preferable for the injections to be preceded by the application of anesthetic creams or be associated with injections of lidocaine [97].

Intralesional steroid therapy may be preceded by a light cryotherapy with liquid nitrogen, 10–15 minutes before injection, to improve the dispersion of the drug in scar tissue and minimize the deposition in the subcutaneous and perilesional tissue [98]. The steroid that is currently most frequently used in the treatment of hypertrophic scars and keloids is triamcinolone acetonide (10–40 mg/mL) [99]. The most common adverse reactions are hypopigmentation, skin atrophy, telangiectasia, and infections [100]. As for injuries to the face, the use of intralesional steroids is recommended for the treatment of individual elements which are particularly bulky and refractory to previous less invasive methods.

#### 4.2.3. Cryotherapy

Cryotherapy with liquid nitrogen can significantly improve the clinical appearance of hypertrophic scars and keloids and also determine their complete regression. 

The low temperatures reached during cryotherapy sessions cause a slowing of blood flow and cause the formation of intraluminal thrombus hesitant to anoxia and tissue necrosis [101]. Age and size of the scar are important factors conditioning the outcome of this technique: younger and smaller scars are most responsive to cryotherapy [102]. Compared with intralesional injections of corticosteroids, cryosurgery is significantly more effective than alternative methods for richly vascularized injuries 12 months younger [103]. During each session of cryotherapy the patient is usually subjected to 2-3 cycles, each lasting less than 25 seconds. Cryotherapy can also be used before each cycle of intralesional injections of steroids to reduce the pain of injection therapy and to facilitate the injection of cortisone, generating a small area of edema at the level of the scar tissue to be treated [98]. Possible adverse reactions are represented by hypo- and hyperpigmentation, skin atrophy, and pain [102]. With regard to localized lesions to the face, the possible outcomes of freezing restrict the use of cryotherapy in these areas, especially in cases where the scars are numerous or for dark phenotypes. Therefore, cryotherapy can be taken into consideration especially for scars located on the trunk or for particularly bulky scars on the face.

#### 4.2.4. Pulsed Dye Laser

The use of lasers for hypertrophic scars and keloids was first proposed by Apfelberg et al. [104] and Castro et al. [105] in the 1980s, and since then more lasers with various wavelengths have been introduced. Unfortunately, laser therapy for hypertrophic scars has had only variable success in the past due to the minimal improvement in a high percent of patients [106–108].

On the contrary, the use of pulsed dye laser (PDL) has provided encouraging results in the treatment of hypertrophic/keloidal scars over the past 10 years. Several studies have been conducted to investigate how the PDL works on hypertrophic/keloidal scars. They have revealed that PDL decreases the number and proliferation of fibroblasts and collagen fibers appear looser and less coarse [109]. Moreover, PDL also produces an increase in MMP-I3 (collagenese-3) activity and a decrease in collagen type III deposition [110]. As a consequence, PDL flattens and decreases the volume of hypertrophic scars [111, 112], improves texture [113], and increases elasticity [114], usually after two to three treatments [115]. Additionally, pruritis and pain within the scars are significantly improved [116]. Besides, no recurrence or worsening of PDL-treated scars occurs during the 4-year followup after cessation of treatment [116]. The most common side effect of the PDL is purpura which can last as long as 7–10 days. Blistering can also occur as well as hypo- and hyperpigmentation which is more likely in darker skinned individuals [117]. Therefore, the ideal candidates for PDL are patients with lighter skin types (Fitzpatrick Types I–III) because less melanin is present to compete with hemoglobin laser energy absorption [118, 119].

#### 4.2.5. Surgery

For the correction of large facial scars, W-plasty seems to be optimal [12]. This therapeutic procedure causes a disruption of the scar which makes the lesion less conspicuous. Especially in facial surgery, autologous skin transplants, namely, full thickness skin transplant or composite fat-skin graft, are another valuable alternative for achieving wound closure with minimal tension. The preferred donor sites for skin graft used for facial defects are the retro- and preauricular sites as well as the neck [120].

#### 4.2.6. Other Approaches

Other treatment options for hypertrophic acne scars and keloids that can be taken into account include elastic compression, intralesional injection of 5-fluorouracil, imiquimod, interferon, radiotherapy, and bleomycin. All these approaches, however, are more effective for the treatment of hypertrophic scars not caused by acne and their use is not recommended due to their impracticality (elastic compression), the lack of clinical experience in the literature (5 FU, interferon, radiotherapy, bleomycin) the lack of efficacy (imiquimod), and the high costs (interferon).

## 5. Conclusion

There are no general guidelines available to optimize acne scar treatment. There are several multiple management options, both medical and surgical, and laser devices are useful in obtaining significant improvement. Further primary research such as randomized controlled trials is needed in order to quantify the benefits and to establish the duration of the effects, the cost-effective ratio of different treatments, and the evaluation of the psychological improvement and the quality of life of these patients.

## Figures and Tables

**Table 1:** Acne scar morphological classification (adapted from [20]).

Acne Scars Subtype	Clinical Features
Icepick	Icepick scars are narrow (<2 mm), deep, sharply marginated epithelial tracts that extend vertically to the deep dermis or subcutaneous tissue.

Rolling	Rolling scars occur from dermal tethering of otherwise relatively normal-appearing skin and are usually wider than 4 to 5 mm. Abnormal fibrous anchoring of the dermis to the subcutis leads to superficial shadowing and a rolling or undulating appearance to the overlying skin.

Boxcar Shallow <3 mm diameter >3 mm diameter	Boxcar scars are round to oval depressions with sharply demarcated vertical edges, similar to varicella scars. They are clinically wider at the surface than icepick scars and do not taper to a point at the base.

Deep <3 mm diameter >3 mm diameter	They may be shallow (0.1–0.5 mm) or deep (≥0.5 mm) and are most often 1.5 to 4.0 mm in diameter.

**Table 2:** Qualitative scarring grading system (adapted from [21]).

Grades of Post Acne Scarring	Level of disease	Clinical features
1	Macular	These scars can be erythematous, hyper- or hypopigmented flat marks. They do not represent a problem of contour like other scar grades but of color.

2	Mild	Mild atrophy or hypertrophy scars that may not be obvious at social distances of 50 cm or greater and may be covered adequately by makeup or the normal shadow of shaved beard hair in men or normal body hair if extrafacial.

3	Moderate	Moderate atrophic or hypertrophic scarring that is obvious at social distances of 50 cm or greater and is not covered easily by makeup or the normal shadow of shaved beard hair in men or body hair if extrafacial, but is still able to be flattened by manual stretching of the skin (if atrophic).

4	Severe	Severe atrophic or hypertrophic scarring that is evident at social distances greater than 50 cm and is not covered easily by makeup or the normal shadow of shaved beard hair in men or body hair if extrafacial and is not able to be flattened by manual stretching of the skin.
